# Proximal‐dominant knee–ankle coordination compensations in football players with chronic ankle instability under high load

**DOI:** 10.1002/jeo2.70443

**Published:** 2025-10-15

**Authors:** Minghua Xian, Jinpeng Lin, Mingfeng Lu, Yongle Lin, Zeyu Li, Guoli Huang, Dongyu Zeng, Yuan Yan, Kele Xie, Kaijun Cai, Wenhan Huang, Liping Li, Yu Zhang

**Affiliations:** ^1^ School of Public Health Shantou University Shantou Guangdong China; ^2^ Department of Orthopedics, Guangdong Provincial People's Hospital (Guangdong Academy of Medical Sciences) Southern Medical University Guangzhou Guangdong China; ^3^ School of Materials Science and Engineering (National Engineering Research Center for Tissue Restoration and Reconstruction) South China University of Technology Guangzhou China; ^4^ The 8Th Clinical Medical College of Guangzhou University of Chinese Medicine Foshan Guangdong China; ^5^ Foshan Hospital of Traditional Chinese Medicine Foshan Guangdong Province China; ^6^ Physical Education Department Guangzhou Vocational School of Tourism and Business Guangzhou Guangdong China; ^7^ Shantou University Medical College Shantou Guangdong China; ^8^ Guangdong Engineering Technology Research Center of functional repair of bone defects and biomaterial, Guangdong Provincial People's Hospital Guangdong Academy of Medical Sciences Guangzhou Guangdong China

**Keywords:** biomechanics, chronic ankle instability, gait, kinematics, variability

## Abstract

**Purpose:**

Chronic ankle instability (CAI) is associated with alterations in lower limb biomechanics. However, little is known about the knee–ankle joint coordination characteristics in individuals with CAI. This study aimed to investigate sagittal plane kinematic differences and knee–ankle coordination strategies in football players with CAI under different locomotor load conditions.

**Methods:**

Forty male football players (CAI: *n* = 20; control: *n* = 20) completed low‐load (walking: mean 3.76 km/h, 3.2–4.8 km/h) and high‐load (running: mean 14.33 km/h, 12.2–16.8 km/h) treadmill tasks. A 3D motion capture system was used to record knee and ankle kinematics. Statistical Parametric Mapping (SPM) was used to analyse group and speed effects. Vector coding (VC) was applied to quantify knee–ankle coordination patterns, categorised into four coupling modes.

**Results:**

Compared with healthy controls, participants with CAI showed significantly reduced ankle dorsiflexion during running at 22%–42% (*p* < 0.001, –4.9° to –11.3°) and 75%–100% (*p* < 0.001, –5.2° to –7.0%) of the gait cycle. Knee flexion was significantly reduced across the entire running cycle (0%–100%, *p* < 0.001, –12.3° to –33.4°). VC analysis revealed particularly transitions toward proximal‐dominant strategies, such as at 43%–46% (*p* < 0.001, *Δ* = 154.46°) and 62%–63% (*p* = 0.008, *Δ* = 9.39°), indicating increased reliance on knee control. Moreover, at 47%–49%, a rigid coupling state was maintained (*p* = 0.003, *Δ* = 170.19°), while 69%–75% of the gait cycle saw a reemergence of distal‐dominant control during swing (*p* = 0.025– < 0.001, *Δ* = 24.06°–48.45°). *Note*: *Δ* refers to the mean difference between groups.

**Conclusion:**

This study revealed significant differences in knee–ankle joint coordination between CAI patients and healthy controls. The findings suggest that CAI‐related motor control adaptations are load‐dependent and follow a distal‐to‐proximal compensatory strategy. These results highlight the importance of incorporating inter‐joint coordination retraining into rehabilitation, particularly under high‐load locomotor scenarios.

**Level of Evidence:**

N/A.

## Graphical abstract

Load‐dependent alterations in knee–ankle coordination in football players with chronic ankle instability: This summarises a study investigating how chronic ankle instability (CAI) affects sagittal plane kinematics and knee–ankle coordination strategies under different loading conditions (walking and running) in male football players. A total of 40 participants (20 CAI, 20 control) completed treadmill‐based gait assessments, and three‐dimensional motion capture was used to record joint angles. Statistical parametric mapping (SPM) and vector coding (VC) analyses were employed to identify joint‐level and inter‐joint coordination differences.

**Abbreviations**
6DOF: six degrees of freedom
CAI: chronic ankle instability
CRP: continuous relative phase
SPM: statistical parametric mapping
VC: vector coding
VCV: vector coding variability

## INTRODUCTION

Chronic ankle instability (CAI) is a common sequela of lateral ankle sprain, characterised by recurrent episodes of 'giving way' and persistent functional impairments [22]. The incidence of CAI is particularly high among athletes, especially football players, accounting for approximately 20%–30% of all sports‐related injuries [11, 16, 40]. Recent studies have suggested that ankle injuries rarely occur in isolation and are often accompanied by compensatory changes in adjacent joints [8, 27, 31, 33, 43]. However, the underlying biomechanical and neuromuscular control mechanisms remain poorly understood. Therefore, exploring the lower‐limb biomechanical and coordination adaptations of individuals with CAI under varying locomotor loads is of great theoretical and clinical importance for guiding rehabilitation and preventing recurrent injury.

Emerging evidence indicates that individuals with CAI exhibit load‐dependent kinematic adaptations across different motor tasks. In high‐load activities such as jumping and landing, these individuals demonstrate increased ankle dorsiflexion, knee flexion, and hip flexion angles, reflecting compensatory mechanisms aimed at enhancing joint stability and shock absorption [4, 24, 46]. In more routine tasks like walking and jogging, CAI participants tend to recruit proximal joints (e.g., the knee) to offset deficits in ankle control [39, 51]. Further studies on jogging have revealed insufficient dorsiflexion during mid‐stance and increased ankle inversion at initial contact in CAI individuals, along with decreased knee flexion moments and elevated plantarflexion moments, suggesting a 'stiffening' propulsion strategy [5, 26]. Additionally, both Drewes et al. [13] and Koldenhoven et al. [26] reported limited dorsiflexion near peak stance, while some studies [10, 34] observed abnormal inversion angles and forefoot progression during loading response. Collectively, these findings support the existence of task‐specific neuromuscular compensation strategies in CAI individuals across varying load conditions.

Previous research has primarily focused on the adaptive behaviour of knee–ankle movement in individuals with CAI under low to moderate load tasks. For instance, Drewes et al. [13, 14] and De Ridder et al. [7] employed continuous relative phase (CRP) analysis during comfortable walking and reported diminished coordination flexibility between the knee and ankle joints in the CAI group. Expanding on this, Lilley et al. [32] demonstrated reduced lower limb coordination in CAI individuals during moderate‐speed running, characterised by a stiffer motor control strategy. And this studies further investigated intra‐joint ankle adaptations during moderate‐ to high‐intensity running, identifying altered angular relationships between the tibia and rearfoot in CAI individuals—particularly a weakened control over rearfoot inversion in the frontal plane [7, 21, 45]. Additionally, Kwon et al. [28, 29] found that during walking tasks, CAI participants tended to adopt an 'anti‐phase abduction/tibial external rotation' strategy, whereas healthy controls more commonly employed an 'anti‐phase plantarflexion/tibial internal rotation' pattern. These findings suggest population‐specific adaptations in knee–ankle coordination across different gait phases.

However, the understanding of knee–ankle coordination in CAI individuals under high‐load conditions remains limited. Particularly in tasks involving rapid running—characterised by high impact and functional demand—there is a lack of systematic investigation into how CAI individuals leverage knee–ankle synergy to maintain stability. Notably, such high‐load scenarios are among the most frequent triggers of ankle injuries. Neglecting the underlying inter‐joint coordination mechanisms may result in incomplete rehabilitation targeting. In this study, we combined sagittal plane kinematic analysis with vector coding (VC) to examine knee–ankle coordination adaptations in football athletes with CAI across different locomotor loads. This integrated approach reflects the theoretical premise that single‐joint kinematics and inter‐joint coordination represent distinct yet complementary dimensions of neuromechanical control. While sagittal joint angles capture local compensations, VC offers a dynamic map of how joints work together throughout the gait cycle. Using SPM [37], we evaluated group‐level dynamic differences throughout the full gait cycle. We hypothesised that CAI participants would demonstrate load‐dependent adaptations in knee–ankle coordination patterns, reflecting compensatory biomechanical strategies in the lower limb. By integrating single‐joint and inter‐joint metrics, this study aims to reveal deeper control impairments not captured by conventional analysis, thereby informing individualised and targeted clinical interventions.

## METHODS

### Participants

**Figure 1:**
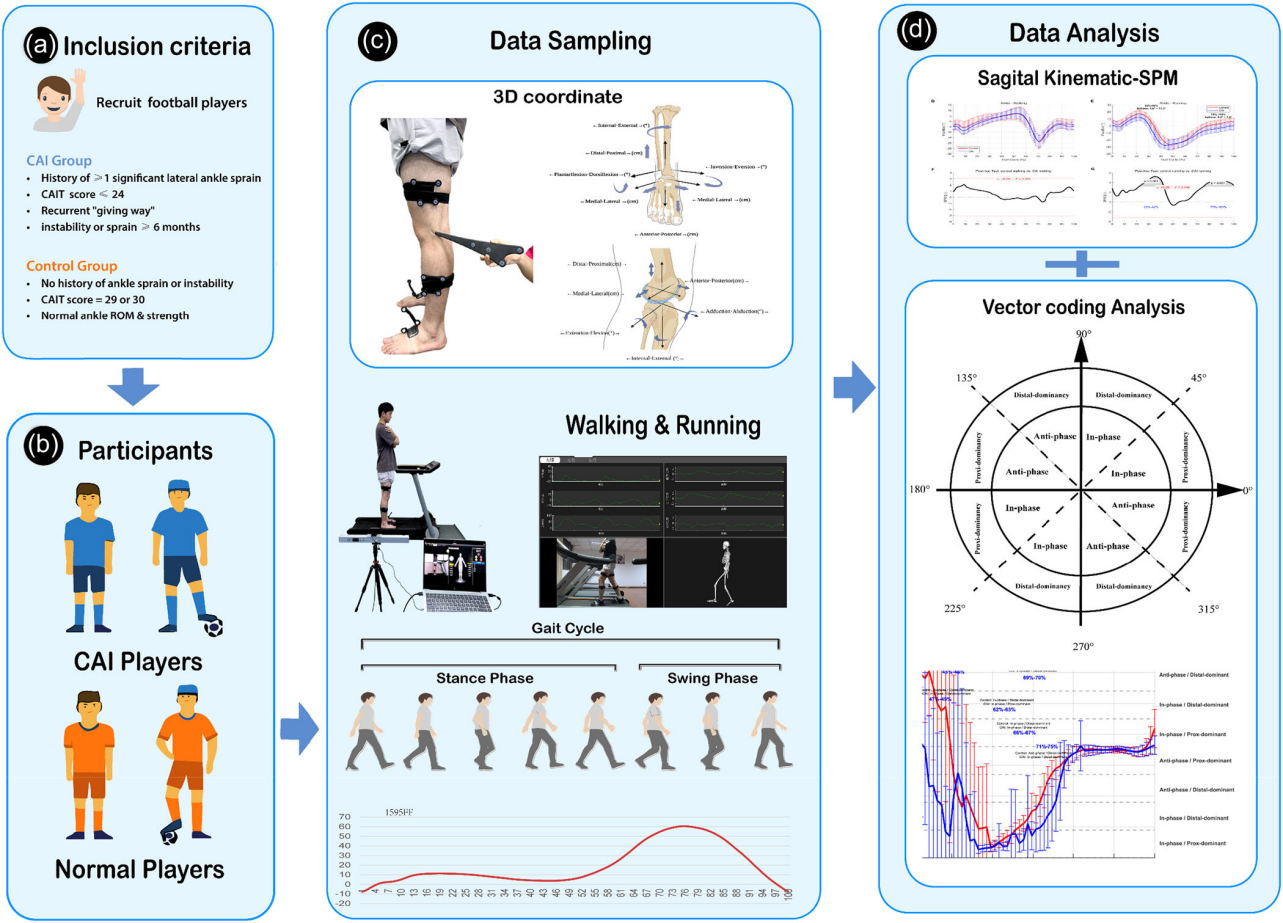
Summary of the study workflow. (a) Criteria for participant inclusion; (b) group assignment of volunteers; (c) gait data collection procedure; (d) kinematic and coordination analysis.

This study was designed as an exploratory biomechanics investigation rather than a hypothesis‐driven clinical trial. Therefore, formal a priori sample size calculation was not conducted. Our group sizes (*n* = 18 per group for final analysis after data quality control) are consistent with numerous peer‐reviewed CAI studies, where typical group sizes ranged from 13 to 28 participants per group [12, 30, 49]. The balanced 1:1 group design adopted here aligns with current standards in coordination and gait analysis literature. All participants were right‐leg dominant and had a minimum of three consecutive years of structured football training experience (see Figure 1a,b).

Inclusion criteria for the CAI group were based on the guidelines of Vicenzino Wikstrom [18, 19] and aligned with the recommendations of the International Ankle Consortium (IAC). All CAI participants had previously been clinically diagnosed with chronic ankle instability at sports medicine clinics based on physical examinations and clinical records. The specific criteria included:

(1) A history of at least one significant lateral ankle sprain that resulted in at least one full day of missed training or physical activity.
(2) A Cumberland Ankle Instability Tool (CAIT) score ≤ 24 for the dominant leg.
(3) Persistent symptoms such as recurrent sprains, giving way, or functional instability lasting more than 6 months.

In line with prior high‐quality studies (e.g., Hertel [22] and Lilley et al. [32]), we did not repeat physical examination procedures (e.g., anterior drawer or talar tilt tests) during field testing sessions, as all CAI participants had received prior orthopaedic diagnoses. Moreover, the objective of this study was to assess functional coordination adaptations, rather than differentiate mechanical versus functional instability through repeated tests.

For the control group, the inclusion criteria were as follows:

(1) No history of ankle injury, instability, or surgery.
(2) Normal ankle range of motion and muscle strength.
(3) A CAIT score of 29 or 30.

Exclusion criteria for all participants, including both CAI and control groups, were identical and included:

(1) any recent lower limb injury (including the hip or knee);
(2) lower limb with surgical history;
(3) diagnosed neurological conditions.

All participants provided written informed consent prior to participation. The study was approved by the Ethics Committee of Guangdong Provincial People's Hospital (Approval No.: 2019‐226H‐1 and conducted in accordance with the Declaration of Helsinki. Demographic and anthropometric characteristics of the participants are presented in Table 1.

**Table 1:** Demographic characteristics of participants in the CAI and control groups.

Variable	CAI group (*n* = 20)	Control group (*n* = 20)	*p*‐Value
Age (years)	18.05 ± 0.76	19.20 ± 2.86	0.090
Height (cm)	173.60 ± 4.07	173.55 ± 6.50	0.977
Weight (kg)	60.65 ± 6.97	64.90 ± 9.03	0.104
CAIT Score	17.80 ± 2.93	29.35 ± 0.33	<0.001***

Abbreviations: CAI, chronic ankle instability; CAIT, Cumberland Ankle Instability Tool.

### Instrumentation

A three‐dimensional motion capture system (Opti_knee, Innomotion Inc., Shanghai, China) was employed to collect six degrees of freedom (6DOF) kinematic data of the knee and ankle joints during treadmill walking and running. This system consists of a dual‐camera infra‐red tracking module (Polaris Spectra, Northern Digital Inc., Canada), high‐resolution optical sensors (Basler aca640‐90uc, Basler AG, Germany), and rigid tracking frames affixed to the femur and tibia [52].

To facilitate accurate anatomical calibration, a handheld digitising probe was used to manually identify and digitise key bony landmarks while participants stood in a neutral position. These anatomical landmarks included the greater trochanter, medial and lateral femoral condyles, tibial tuberosity, fibular head, and medial and lateral malleoli. These points were subsequently used to define subject‐specific local coordinate systems for motion analysis (see Figure 1c).

The cameras were arranged in a fixed configuration, ensuring spatial accuracy within a 10 m² working area. The capture volume had a field of view of approximately 2 × 2 m, with optimal detection occurring within a 2–3 m range. During dynamic trials, motion data were recorded continuously at 60 Hz for 30‐s intervals. Multiple gait cycles were collected, and average joint kinematic values across cycles were computed to enhance measurement reliability.

Based on principles adapted from surgical navigation, the system delivers sub‐millimetre positioning accuracy (0.3 mm RMS), with translational and rotational accuracies below 0.9 mm and 1.3°, respectively. This ensures reliable quantification of joint motion in a real‐time, non‐clinical environment [15, 47].

### Test procedure

All participants completed treadmill‐based gait assessments under two speed conditions: (1) walking at a self‐selected comfortable speed (mean: 3.76 km/h; range: 3.2–4.8 km/h) and (2) running at the fastest speed they could sustainably perform on a treadmill (mean: 14.33 km/h; range: 12.2–16.8 km/h). To ensure safety and consistency, the testing order was fixed for all participants: walking trials were always conducted prior to running. Both tasks were performed on the same treadmill to minimise variability associated with equipment differences.

Prior to testing, motion capture markers were affixed to the participants, followed by a standardised standing calibration to establish anatomical reference positions. During each gait task, participants were instructed to maintain a steady pace for 30 seconds, during which valid gait cycles were continuously recorded. All participants completed both conditions on the same day, with sufficient rest between tasks to prevent fatigue‐related bias.

### Test conditions

(1) Comfortable‐speed walking: Participants walked at a self‐selected comfortable pace (mean: 3.76 km/h; range: 3.2–4.8 km/h), simulating a habitual, low‐load gait. This speed range corresponds well with normative values for healthy adults (127.2–146.2 cm/s, approximately 4.6–5.3 km/h) reported by Bohannon et al. [2], thereby supporting its representativeness.
(2) High‐speed running: Participants then ran at the highest speed they could sustainably maintain on the treadmill (mean: 14.33 km/h; range: 12.2–16.8 km/h), designed to simulate high‐demand functional loading conditions. This velocity significantly exceeds the maximal walking speed (253.3 cm/s, ~9.1 km/h) reported by Bohannon et al. [2], qualifying it as a high‐load locomotor task.

The use of individualised speed settings enabled a more accurate reflection of each participant's functional capacity while avoiding task difficulty bias associated with uniform speed assignments. Prior studies have shown that increased locomotor speed is associated with greater joint loading, reflected by elevated flexion‐extension joint moments and power outputs at the knee and ankle joints [3, 6, 42]. Accordingly, this design enhances ecological validity for assessing the dynamic compensatory mechanisms in individuals with CAI under real‐world high‐load conditions.

### Data processing

Kinematic data of the knee and ankle joints were collected in six degrees of freedom (6DOF). For the current analysis, sagittal plane joint angles (i.e., flexion‐extension) of the dominant leg were extracted. Each gait cycle was time‐normalised to 100 data points (0%–100%), and five valid gait cycles were selected per task for each participant to ensure consistency. A complete gait cycle was defined as the interval between two successive heel strikes of the same foot.

To investigate inter‐joint coordination between the knee and ankle, an enhanced VC technique was employed based on the method described by Needham et al. [36]. Specifically, angle–angle plots were constructed using the sagittal plane angles of the proximal (knee) and distal (ankle) joints, as per Lilley et al. [32].

VC was selected over other coordination measures such as CRP due to its methodological advantages in capturing joint dominance and discrete coordination modes. VC was originally derived from the dynamical systems framework of Sparrow et al. [44], and later refined by Hamill et al. [20]and Needham et al. [35, 36] for human gait. Compared to CRP, VC does not require phase extraction via Hilbert transforms or velocity computation, and has been shown to exhibit greater robustness in CAI populations. Recent studies including Samson et al. [41], Silvernail et al. [17], and Wasser et al. [48] have supported VC's enhanced sensitivity in detecting dynamic coordination changes, particularly in pathological gait.

The coupling angle (i.e., VC, vector coding angle) was defined as the instantaneous orientation of the vector connecting two adjacent time points in the angle–angle space. The VC was calculated using the following formula:

$$\theta_{i}=\mathrm{mod}(\tan^{-1}(\frac{∆Y_{i}}{∆x_{i}})\times \frac{180}{\pi },360),$$

where $∆X_{i}=\;X_{i+1}-X_{i}$ represents the change in proximal joint (knee) angle, and $∆Y_{i}=\;Y_{i+1}-Y_{i}$ represents the change in distal joint (ankle) angle between consecutive time points. The modulus function ensures that the resulting angle is within the range of [0°, 360°]. This method does not require velocity calculations and allows for direct interpretation of coordination patterns.

To evaluate average coupling behaviour across multiple strides, circular statistics were applied following Batschelet [1]. At each percentage point of the gait cycle, horizontal and vertical vector components were averaged across the selected gait cycles. The mean coupling angle $\bar{\theta }$ was then computed using:

$$\bar{\theta }=\mathrm{mod}(\tan^{-1}(\frac{\sin\sum \left(\theta_{i}\right)}{\cos\sum \left(\theta_{i}\right)})\times \frac{180}{\pi },360).$$

**Figure 2:**
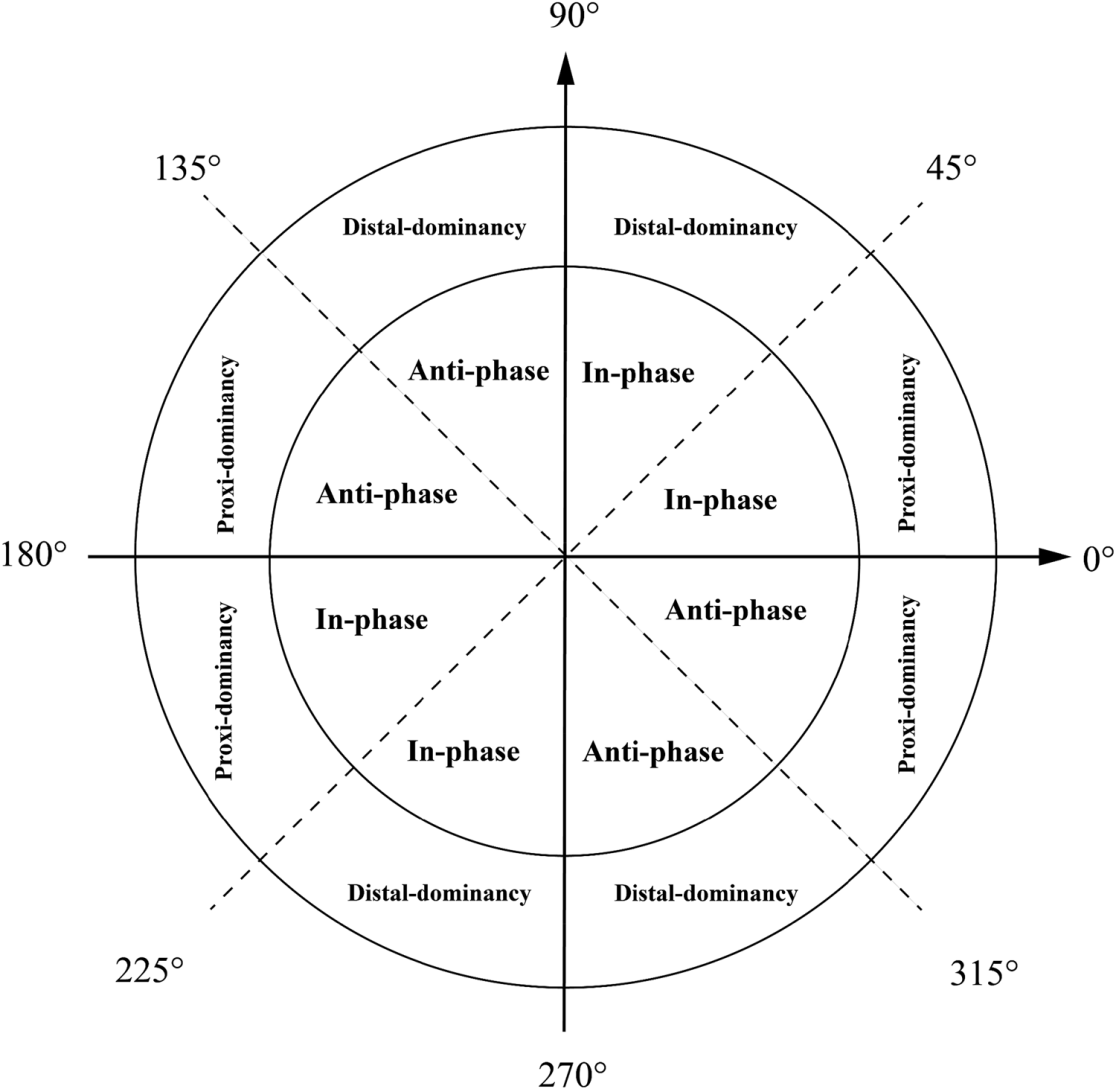
Classification of coordination pattern. The coupling angle was categorised into four coordination patterns based on phase (in‐phase vs. anti‐phase) and joint dominance (proximal‐dominant vs. distal‐dominant).

Subsequently, coupling angles were classified into four discrete coordination patterns based on the framework proposed by Needham et al. [36], as illustrated in Figure 2. This classification considered both motion phase (in‐phase vs. anti‐phase) and joint dominance (proximal‐dominant vs. distal‐dominant), offering clinically meaningful insights into compensatory strategies. All VC calculations and pattern classifications were performed using custom scripts developed in MATLAB R2023b.

### Statistical analysis

Independent sample *t*‐tests were used to compare demographic characteristics (age, height, weight, and body mass index) between the CAI and control groups. Normality was assessed using the Shapiro–Wilk test, and homogeneity of variance was evaluated with Levene's test. A significance threshold of *p* < 0.05 was applied.

To investigate gait differences at the joint level, repeated‐measures two‐way analysis of variance (ANOVA) within the Statistical Parametric Mapping (SPM) framework was performed on the sagittal plane kinematic time series of the knee and ankle joints. The two factors included group (CAI vs. control) and speed condition (walking at 3 km/h vs. running at 12 km/h). This time‐continuous approach allowed for analysis of the entire gait cycle without losing temporal resolution.

When significant main effects or interactions were observed, post‐hoc independent *t*‐tests were conducted within the SPM framework to identify specific phases of the gait cycle where differences occurred. A similar SPM‐based two‐way ANOVA was applied to the VC data to examine coordination differences, followed by post hoc *t*‐tests to detect significant group‐specific coupling alterations during distinct gait phases.

All SPM analyses were performed using the open‐source *spm1d* package [37, 38] (version M.0.4.10; www.spm1d.org) in MATLAB R2023b. Standard statistical procedures, including demographic comparisons, were conducted in R version 4.3.1. Statistical significance was defined as *p* < 0.05. When appropriate, Bonferroni correction was applied to adjust for multiple comparisons.

To ensure clarity and conciseness in the main text, only representative results of the SPM analysis are presented. Complete post hoc SPM‐t results, including detailed time intervals of significant differences, are provided in Supporting Information: Figures 1–4) to facilitate transparency and reproducibility.

## RESULTS

### Sagittal plane joint kinematics

**Figure 3:**
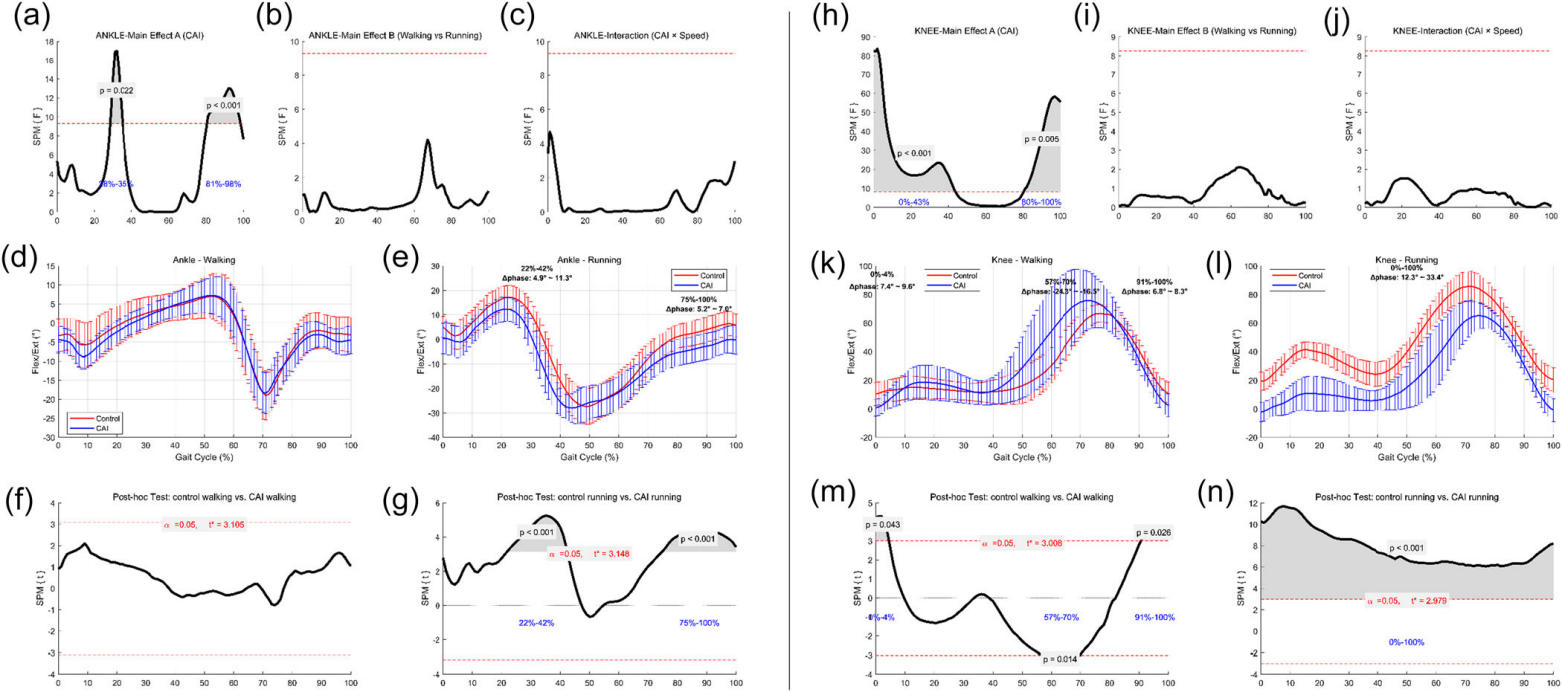
Sagittal plane joint kinematics and statistical results. Figure 3 illustrates the sagittal plane joint kinematics and corresponding statistical results for both the ankle (left column) and knee (right column) joints across walking and running conditions. ‐ Row 1 (a–c, h–j): SPM{F} curves from the two‐way ANOVA showing the main effects of Group (a, h), Speed (b, i), and the Group × Speed interaction (c, j). ‐ Row 2 (d–e, k–l): Group‐averaged flexion‐extension trajectories for walking and running, with shaded areas representing standard deviations. ‐ Row 3 (f–g, m–n): Post hoc SPMt results comparing CAI and control groups under walking (f, m) and running (g, n). Significant intervals (*p* < 0.05) are shaded in grey, highlighting time periods of between‐group differences in joint motion. ANOVA, analysis of variance; SPM, Statistical Parametric Mapping.

As illustrated in Figure 3, the flexion‐extension profiles of the ankle and knee joints are presented in three rows. The figure is vertically divided by a dashed line into two panels: the left panel (a–g) displays results for the ankle joint, and the right panel (h–n) presents findings for the knee joint.

### Ankle joint kinematics

During walking, a significant group main effect was observed between 22% and 42% of the gait cycle (ANOVA‐A, *p* < 0.001; range: 4.9°–11.3°). This overlapped with the post‐hoc SPM{t} result, which indicated significant group differences from 28% to 35% (*p* = 0.022), suggesting that the differences originated primarily from group‐level effects.

During running, the group effect remained significant from 81% to 98% of the gait cycle (ANOVA‐A, *p* < 0.001), which also corresponded with the post hoc SPM{t} result identifying a significant difference from 75% to 100% (*p* < 0.001; range: 5.2°–7.0°). These overlapping periods confirmed that the observed kinematic differences were primarily attributable to CAI, rather than speed‐induced effects.

### Knee joint kinematics

For the knee joint during walking, a significant group effect was found during 0%–43% (*p* < 0.001) and 80%–100% (*p* = 0.005) of the gait cycle. Corresponding post hoc SPM{t} results revealed significant differences in 0%–4% and 91%–100% intervals (*p* = 0.026; range: –24.3° to –16.5°), indicating that part of the kinematic differences stemmed from group effects. However, certain significant SPM{t} regions (e.g., 57%–70%, *p* = 0.014; range: 6.8°–8.3°) did not align with ANOVA findings, suggesting that walking speed modulated these phase‐specific differences.

During running, group‐level differences were present throughout the entire gait cycle, as indicated by ANOVA‐A significance in 0%–43% and 80%–100%, and post‐hoc SPM{t} findings showing significant group differences across 0%–100%.

Table 2 provides detailed statistics for these intervals. For instance, during walking, knee flexion differences reached 6.8°–9.6° in the 91%–100% interval, whereas during running, the between‐group differences expanded to 12.3°–33.4°, reflecting more pronounced compensatory mechanisms under high‐load conditions.

**Table 2:** Significant differences in flexion/extension angles between control and CAI (based on SPM analysis).

Joint	Condition	Analysis type	Significant interval (%)	*p*‐Value	ΔPhase (°)
Ankle		ANOVA‐Effect A	28%–35%	*p* = 0.022	–
		ANOVA‐Effect A	81%–98%	*p* < 0.001	–
	Running	t‐test	22%–42%	*p* < 0.001	4.9–11.3
		t‐test	75%–100%	*p* < 0.001	5.2–7.0
Knee		ANOVA‐Effect A	0%–43%	*p* < 0.001	–
		ANOVA‐Effect A	80%–100%	*p* = 0.005	–
	Walking	t‐test	0%–4%	*p* = 0.043	7.4–9.6
		t‐test	57%–70%	*p* = 0.014	−24.3 to −16.5
		t‐test	91%–100%	*p* = 0.026	6.8–8.3
	Running	t‐test	0%–100%	*p* < 0.001	–

*Note*: This table provides detailed statistics for these intervals. For instance, during walking, knee flexion differences reached 6.8°–9.6° in the 91%–100% interval, whereas during running, the between‐group differences expanded to 12.3°–33.4°, reflecting more pronounced compensatory mechanisms under high‐load conditions.

Abbreviations: CAI, chronic ankle instability; CAIT, Cumberland Ankle Instability Tool.

### Summary

Overall, significant sagittal plane kinematic differences between the CAI and control groups were observed in both ankle and knee joints. Notably, ankle joint differences were primarily evident during high‐load running, while knee joint differences were present under both walking and running conditions, indicating a more consistent role of the knee in compensatory gait adaptations. These results validate the scientific rationale for investigating knee–ankle compensatory strategies in individuals with CAI.

### VC coupling pattern

**Figure 4:**
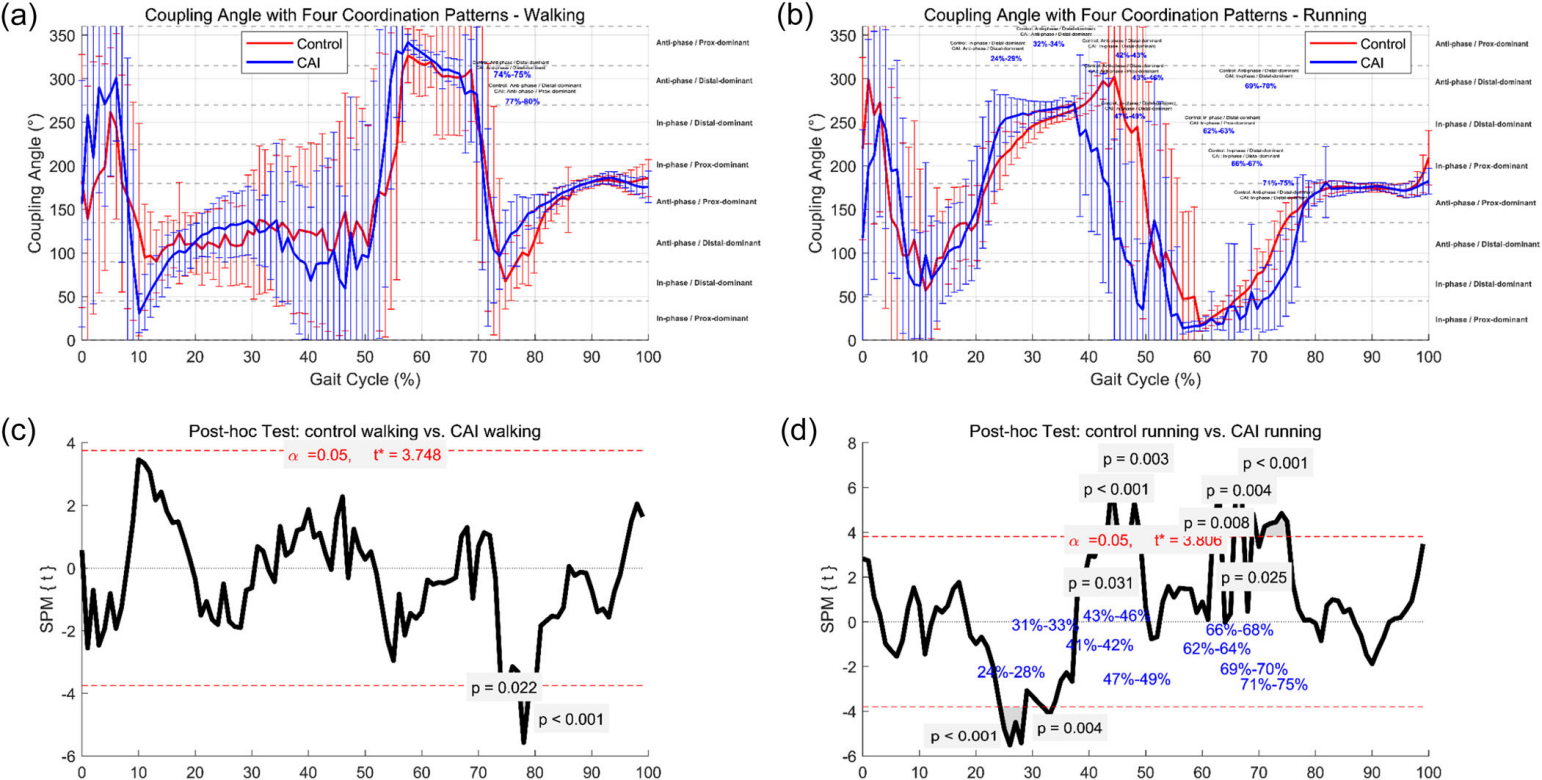
Coupling angle patterns and statistical comparisons between groups during walking and running. (a–b) Show mean coupling angle curves for walking and running, with shaded bands indicating standard deviations and significant gait intervals annotated alongside coordination quadrant classifications. (c–d) Illustrate the post‐hoc SPM{t} results highlighting significant group differences between the CAI and control groups. The red dashed lines indicate the critical threshold (α = 0.05).

Beyond joint angle differences, VC analysis further revealed inter‐joint coordination strategies between the knee and ankle. Figure 4 presents the coupling angle curves and post‐hoc SPM comparisons under both walking and running conditions. (a–b) show mean coupling angle curves for walking and running, with shaded bands indicating standard deviations and significant gait intervals annotated alongside coordination quadrant classifications. Panels (c–d) illustrate the post‐hoc SPM{t} results highlighting significant group differences between the CAI and control groups. The red dashed lines indicate the critical threshold (*α* = 0.05).

### Walking

During walking, two distinct gait intervals exhibited significant group differences. At 74%–75% of the gait cycle, although both groups maintained the same coordination pattern ('anti‐phase/distal‐dominant'), a significant difference in coupling angle was observed (*p* = 0.022; *Δ* = –24.92°), corresponding to late stance. This suggests that even without a change in coordination pattern, CAI participants may engage altered neuromuscular timing or joint stiffening strategies. At 77%–80%, the CAI group exhibited a transition from “anti‐phase/distal‐dominant” to “anti‐phase/proximal‐dominant” (*p* < 0.001; *Δ* = –38.40°), indicating a compensatory shift in control from the ankle to the knee during push‐off.

### Running

Under the high‐load running condition, a broader range of gait intervals demonstrated significant differences in knee–ankle coordination strategies between the CAI and control groups. From mid‐stance to early swing, multiple transitions and angular deviations were observed. At 24%–29% of the gait cycle, a transition from 'in‐phase/distal‐dominant' to “anti‐phase/distal‐dominant” was detected (*p* < 0.001; *Δ* = –33.66°), reflecting decreased synchrony and potential instability during early stance. At 32%–34%, although the coordination pattern remained unchanged, the significant angular deviation (*p* = 0.004; *Δ* = –11.54°) indicated subtle neuromotor adaptation during mid‐stance. A subsequent return to a more stable 'in‐phase/distal‐dominant' pattern occurred at 42%–43% (*p* = 0.031; *Δ* = 88.52°). From 43%–46%, a distinct transition toward a “anti‐phase/proximal‐dominant” strategy was observed (*p* < 0.001; *Δ* = 154.46°), suggesting increased compensatory reliance on knee control under high mechanical demand. This proximal shift continued at 62%–63% (*p* = 0.008; *Δ* = 9.39°) and remained evident at 66%–67% (*p* = 0.004; *Δ* = 19.70°), likely reflecting sustained proximal engagement during late stance. Additionally, at 47%–49%, while both groups preserved the 'in‐phase/distal‐dominant' pattern, a significant difference in coupling angle persisted (*p* = 0.003; *Δ* = 170.19°), indicating increased coupling rigidity. Finally, during the swing phase (69%–70% and 71%–75%), the CAI group exhibited a transition from 'anti‐phase/distal‐dominant' to 'in‐phase/distal‐dominant' (*p* = 0.025 and < 0.001; *Δ* = 24.06° and 48.45°, respectively), suggesting a late‐phase attempt to restore distal control.

Table 3 summarises all gait intervals showing significant between‐group differences in coupling angles, including the mean difference (*Δ*) and the corresponding classification of coordination mode transitions (or maintenance).

**Table 3:** Classification of VC coupling patterns and mean differences between control and CAI groups across significant gait cycle intervals.

	Significant interval (%)	Control coupling pattern	CAI coupling pattern	*p*‐Value	Mean difference (Control – CAI; °)
**W**alking‐VC	74–75	Anti‐phase/distal‐dominant	Anti‐phase/distal‐dominant	0.022	−24.9
	77–80	Anti‐phase/distal‐dominant	Anti‐phase/prox‐dominant	<0.001	−38.4
Running‐VC	24–29	In‐phase/distal‐dominant	Anti‐phase/distal‐dominant	<0.001	−33.7
	32–34	Anti‐phase/distal‐dominant	Anti‐phase/distal‐dominant	0.004	−11.5
	42–43	Anti‐phase/distal‐dominant	In‐phase/distal‐dominant	0.031	88.5
	43–46	Anti‐phase/distal‐dominant	Anti‐phase/prox‐dominant	<0.001	154.5
	47–49	In‐phase/distal‐dominant	In‐phase/distal‐dominant	0.003	170.2
	62–63	In‐phase/distal‐dominant	In‐phase/prox‐dominant	0.008	9.4
	66–67	In‐phase/distal‐dominant	In‐phase/distal‐dominant	0.004	19.7
	69–70	Anti‐phase/distal‐dominant	In‐phase/distal‐dominant	0.025	24.1
	71–75	Anti‐phase/distal‐dominant	In‐phase/distal‐dominant	<0.001	48.5

Abbreviations: CAI, chronic ankle instability; VC, cector coding.

### Summary

These findings suggest that individuals with CAI adopt more proximal‐dominant and phase‐adjusted coordination strategies under high‐load conditions. Notably, many of these coordination changes occurred in gait phases where sagittal plane joint angles showed no significant group‐level differences, underscoring the unique sensitivity of VC in detecting compensatory neuromuscular mechanisms.

In this study, we focused on sagittal–sagittal coordination (e.g., knee flexion–ankle dorsiflexion) as our primary vector coding (VC) metric, given its biomechanical relevance to propulsion and shock absorption phases of gait. To explore potential cross‐planar interactions, we additionally analysed two common coupling pairs: knee sagittal – ankle frontal (KS–AF) and knee sagittal – ankle transverse (KS–AT), following approaches by Jeong et al. [25] and Lilley et al. [32]. However, neither combination yielded statistically significant differences across conditions, whereas the sagittal–sagittal coupling revealed robust load‐dependent effects. Therefore, sagittal coupling was prioritised for further discussion to reflect both its mechanistic importance and observed sensitivity. The results of the KS–AF and KS–AT coupling analyses are provided in Supporting Information: Figures 5–8) for reference.

## DISCUSSION

This study VC analysis to investigate knee–ankle coordination strategies in football athletes with CAI under both walking and running conditions. The primary findings revealed that CAI individuals exhibited reduced dorsiflexion and knee flexion under high‐load conditions, accompanied by a coordination shift from distal‐dominant to proximal‐dominant patterns. Notably, several transitions in coupling strategies occurred without corresponding joint angle differences, underscoring the superior sensitivity of coordination‐based metrics over single‐joint kinematics.

To capture both local and global adaptations, we employed an integrated framework combining sagittal plane joint angle analysis with inter‐joint coordination assessment via VC. This approach reflects the theoretical premise that joint‐specific kinematics and inter‐joint coordination represent distinct yet complementary dimensions of neuromechanical control. Although only sagittal–sagittal coordination was emphasised, this choice aligns with its well‐established role in gait propulsion and shock absorption. Additional cross‐planar coupling analyses (e.g., KS–AF and KS–AT) did not reveal significant differences and are presented in the appendix. These findings highlight the clinical relevance of incorporating coordination retraining into rehabilitation protocols—particularly under high‐load tasks—to address functional instability more effectively.

### Knee–ankle compensatory coordination during walking in CAI

During the walking task, although no significant differences were observed in sagittal plane joint angles of the knee or ankle within the 74%–80% interval of the gait cycle, VC analysis revealed alterations in coordination strategies. Specifically, the CAI group exhibited a transition from an 'anti‐phase/distal‐dominant' to an “anti‐phase/proximal‐dominant” pattern between 77%–80%, suggesting that even in the absence of joint angle differences, CAI individuals may adapt inter‐joint coordination to maintain dynamic stability.

This observation aligns with findings by Kwon et al. [29], who reported significant between‐group differences in coupling angles despite the absence of segmental angle differences. Their subsequent work [28] emphasised that VC analysis is more sensitive than joint angle analysis in detecting alterations in motor control strategies among CAI individuals. Our study, through the integration of sagittal plane SPM and VC‐SPM analyses, further supports the utility of coordination metrics in revealing latent compensatory mechanisms, particularly in gait phases characterised by minimal joint kinematic deviation.

Earlier studies on CAI‐related gait abnormalities during treadmill or overground walking [5, 9] also support this interpretation. While our current analysis did not reveal significant sagittal plane differences in joint angles during mid‐stance to toe‐off phases, the VC analysis did uncover a transition in coordination pattern, suggesting that neuromuscular control adaptations may occur independently of ROM alterations.

### Coordination reorganisation induced by high‐load running

Under high‐load running conditions, the CAI group exhibited clear differences in both sagittal joint angles and coordination patterns. Specifically, ankle dorsiflexion and knee flexion angles were significantly lower than those in the control group throughout the gait cycle (ankle: 4.9°–11.3°; knee: 12.3°–33.4°). Although joint moments were not measured in the present study, prior research has suggested that reduced ankle motion may be accompanied by increased knee joint loading [50]. Additionally, Doherty et al. [12] observed decreased knee flexion moments prior to toe‐off in CAI participants, which was interpreted as a stiffer neuromuscular control strategy to cope with increased load demands.

Importantly, the time interval showing significant ankle angle differences (22%–42%) overlapped with the VC‐detected coordination shifts (24%–29%, 32%–34%), indicating concurrent compensation at both the kinematic and inter‐joint coordination levels. In contrast, during 75%–100% of the gait cycle, ankle angle differences were observed without any coordination changes, suggesting that compensation during this phase was achieved through isolated joint adjustments. Conversely, intervals such as 43%–46% and 66%–67% displayed coordination changes without joint angle differences, implying that compensation may have shifted to a control strategy level.

Notably, from 62% to 63% of the gait cycle, the CAI group transitioned from an “in‐phase/distal‐dominant” pattern to a 'proximal‐dominant' strategy, subsequently maintaining an anti‐phase mode. This observation is consistent with findings by Drewes et al. [13] and De Ridder et al. [7], which indicated reduced coupling stability during late stance. Kwon et al. [29] further reported directional asymmetries in coupling patterns among CAI individuals: dorsiflexion was coupled with tibial external rotation in the CAI group, while plantarflexion was coupled with tibial internal rotation in controls. Although both were anti‐phase patterns, they occurred in opposite directions, suggesting a compensatory mechanism via directional adjustment of joint interactions.

Moreover, the CAI group frequently exhibited proximal shifts in VC patterns (e.g., 24%–29%, 43%–46%, and 62%–63%), indicating increased knee‐dominant control. Lilley et al. [32] also found that CAI participants demonstrated reduced vector coding variability (VCV) during running, reflecting a more rigid but less adaptable control strategy. While reduced variability is not inherently maladaptive, it may compromise adaptability in dynamic environments and increase the risk of injury.

### Load‐dependent compensation mechanisms

The findings of this study highlight the load‐dependent nature of compensatory strategies in individuals with CAI. Specifically, sagittal kinematic differences at the ankle were primarily observed under high‐load conditions such as running, whereas the knee joint exhibited group‐level differences during both walking and running, suggesting its stabilising role in gait coordination. VC analysis further revealed that CAI individuals were more likely to exhibit shifts from distal‐dominant to proximal‐dominant coordination patterns during high‐demand tasks, particularly in mid‐to‐late stance—the critical phase for maintaining postural stability and generating push‐off power. For example, during running, the CAI group displayed a significant transition from an 'anti‐phase/distal‐dominant' to an 'anti‐phase/proximal‐dominant' pattern between 43%–46% of the gait cycle (*Δ* = 154.46°), indicating an increased reliance on knee control.

These coupling changes are indirectly supported by findings from kinetic studies. Jang et al. [23] reported that even under moderate‐speed walking, CAI individuals showed significant reductions in ankle joint moments and power output during peak dorsiflexion and push‐off phases. Doherty et al. [12] further observed that CAI participants exhibited reduced knee flexion moments prior to toe‐off, suggesting a stiffer neuromuscular control strategy to accommodate high‐load demands during push‐off. Although joint kinetics were not directly measured in this study, such evidence provides a neuro‐biomechanical rationale for the coupling pattern shifts observed in CAI individuals during high‐speed running.

### Clinical and rehabilitation implications

The findings of this study underscore the clinical relevance of evaluating inter‐joint coordination in individuals with CAI, particularly under conditions of increased mechanical demand. Traditional rehabilitation programmes often focus on restoring joint range of motion (ROM) or isolated muscle strength, potentially overlooking subtle yet critical deficits in dynamic joint coordination. In contrast, our results suggest that knee–ankle coupling retraining should be systematically incorporated into clinical protocols, especially for athletes exposed to rapid directional changes and high‐impact activities.

VC‐based assessments may assist clinicians in identifying coordination impairments even when joint kinematics appear normal. Rehabilitation interventions could include perturbation‐based treadmill walking, joint coordination biofeedback, or phase‐specific proprioceptive training that simulates high‐load gait conditions. Neuromuscular retraining aimed at restoring optimal coordination patterns—such as correcting maladaptive anti‐phase or proximal‐dominant responses—may lead to more personalised and effective rehabilitation outcomes, as well as reduce the risk of re‐injury.

### Limitations and future directions

This study focused exclusively on sagittal plane coordination between the knee and ankle joints. However, compensatory adaptations in CAI likely involve multidimensional changes, including alterations in frontal (e.g., inversion–eversion) and transverse (e.g., tibial rotation) planes, particularly given that many injury mechanisms occur in multiplanar contexts. Future research should extend VC analysis to incorporate multiplanar dimensions and integrate joint moment assessments to more comprehensively capture the relationship between joint loading and neuromechanical adaptation.

Another limitation lies in the use of a discrete, binary classification system for identifying coordination patterns. While this approach offers good interpretability, it may oversimplify the continuous nature of joint coordination. Emerging techniques such as continuous coupling angle representation, vector field clustering, or machine learning–based classification frameworks could improve resolution and clinical applicability in coordination analysis.

Moreover, although the current sample size was sufficient for preliminary investigation, generalisability remains to be verified. Future studies should be conducted with larger, multicenter cohorts involving diverse athletic populations, and incorporate longitudinal tracking before and after intervention to evaluate whether VC patterns serve as objective indicators of rehabilitation progress and injury risk stratification.

Finally, this study was designed to investigate functional compensation patterns during gait in individuals with CAI, rather than directly assess underlying structural lesions. Although all CAI participants had prior clinical diagnoses and typical symptomatic histories, we did not incorporate imaging techniques (e.g., MRI or ultrasonography) to evaluate specific anatomical structures such as the calcaneofibular ligament (CFL) or subtalar joint stability. Given that altered mechanical integrity in these structures may underlie the observed neuromechanical compensations, future studies should integrate quantitative imaging and dynamic gait analysis to bridge the gap between structural pathology and functional adaptations.

## CONCLUSION

This study demonstrated that individuals with CAI exhibit distinct knee–ankle coordination patterns under varying locomotor loads. Notably, CAI participants showed task‐specific coordination shifts during high‐load running, particularly characterised by transitions toward proximal‐dominant and anti‐phase coupling patterns. These findings suggest that CAI‐related motor control adaptations are both load‐dependent and segmentally distributed, underscoring the importance of incorporating inter‐joint coordination retraining into rehabilitation strategies.

## AUTHOR CONTRIBUTIONS

Minghua Xian, Jinpeng Lin, and Mingfeng Lu contributed equally to this work and share first authorship. All authors contributed to study design, data collection, analysis, and manuscript revision. Yu Zhang, Wenhan Huang, and Liping Li are the corresponding authors and supervised the study.

## CONFLICT OF INTEREST STATEMENT

The authors declare no conflict of interest.

## ETHICS STATEMENT

The study was approved by the Ethics Committee of Guangdong Provincial People's Hospital (Approval No: 2019‐226H‐1). Written informed consent was obtained from all adult participants. For minor participants, informed consent was signed by their legal guardians.

## Supporting information

supmat.

## Data Availability

The data supporting the findings of this study are available from the corresponding author upon reasonable request.
