# Supplementary material for: Proximal‐dominant knee–ankle coordination compensations in football players with chronic ankle instability under high load
Source: J Exp Orthop. 2025 Oct 15;12(4):e70443. doi: 10.1002/jeo2.70443 (PMC12527221; doi:10.1002/jeo2.70443)
Supplement: Supplementary file 1 — supmat. [file JEO2-12-e70443-s001.docx]

**Extra supplement**

**Supplementary: Line 231**

**Supplement_Figure1_Ankle_WalkRun.tif**

Comparison of ankle flexion-extension angles between walking and running conditions.

(a–b) Mean ± SD curves for ankle flexion-extension in control and CAI groups, respectively, under walking and running conditions;

(c–d) Post-hoc SPM-t tests showing significant within-group differences between walking and running.
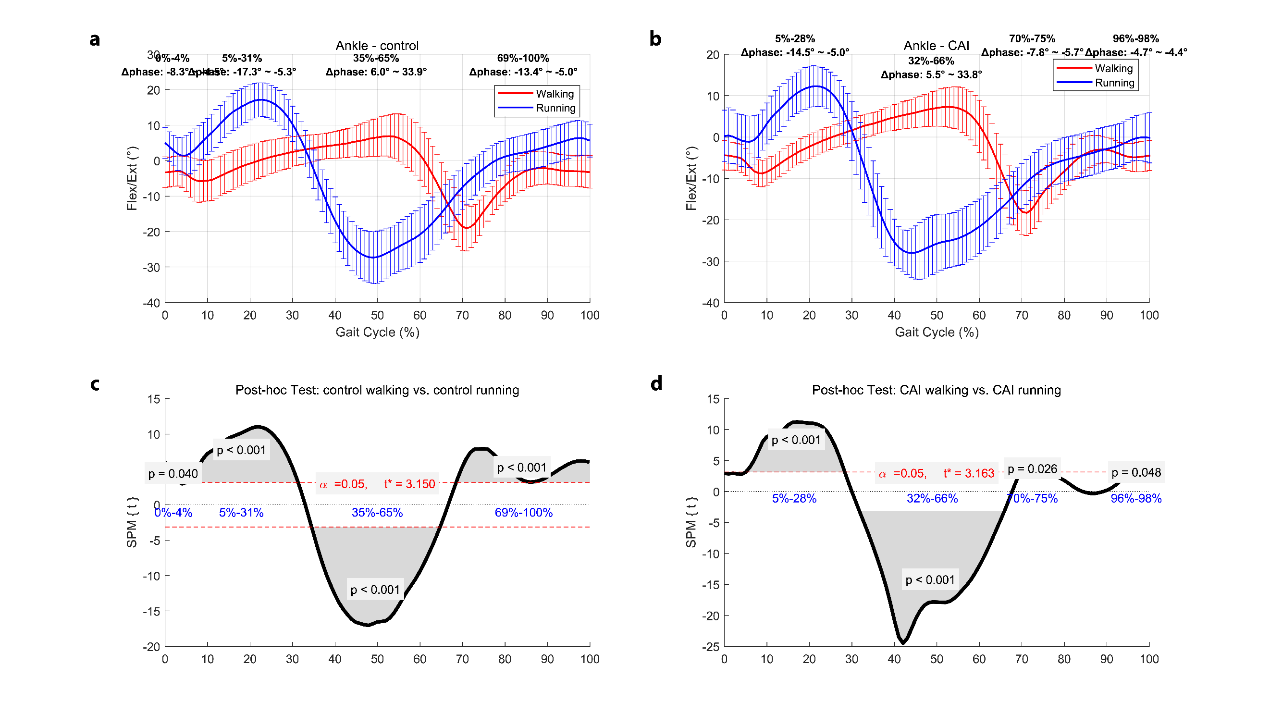


**Supplement_Figure2_Ankle_GroupCompare.tif**

Comparison of ankle flexion-extension between CAI and control groups across pooled gait conditions (walking + running).

(a–b) Mean ± SD curves of ankle flexion-extension for CAI and control groups;

(c–d) SPM-t tests showing significant group differences across combined conditions.
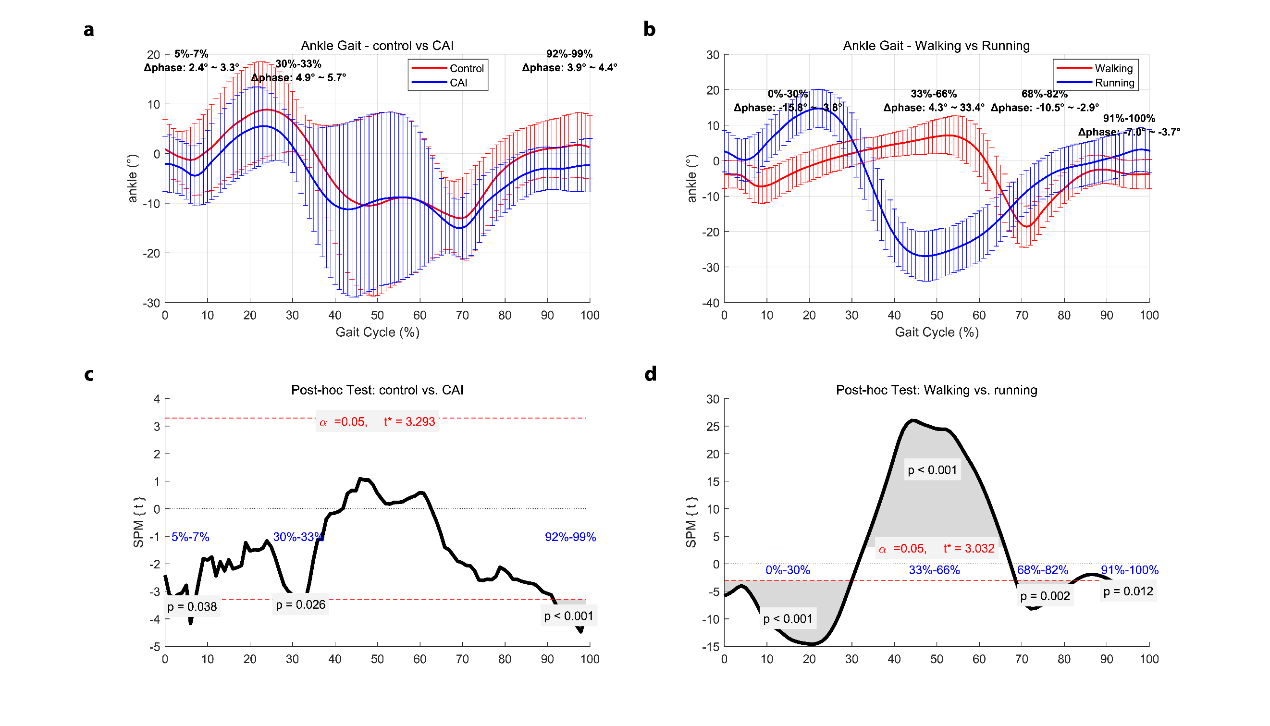


**Supplement_Figure3_Knee_WalkRun.tif**

Comparison of knee flexion-extension angles between walking and running conditions.

(a–b) Mean ± SD curves of knee flexion-extension in control and CAI groups under walking and running;

(c–d) Within-group SPM-t tests identifying significant differences.
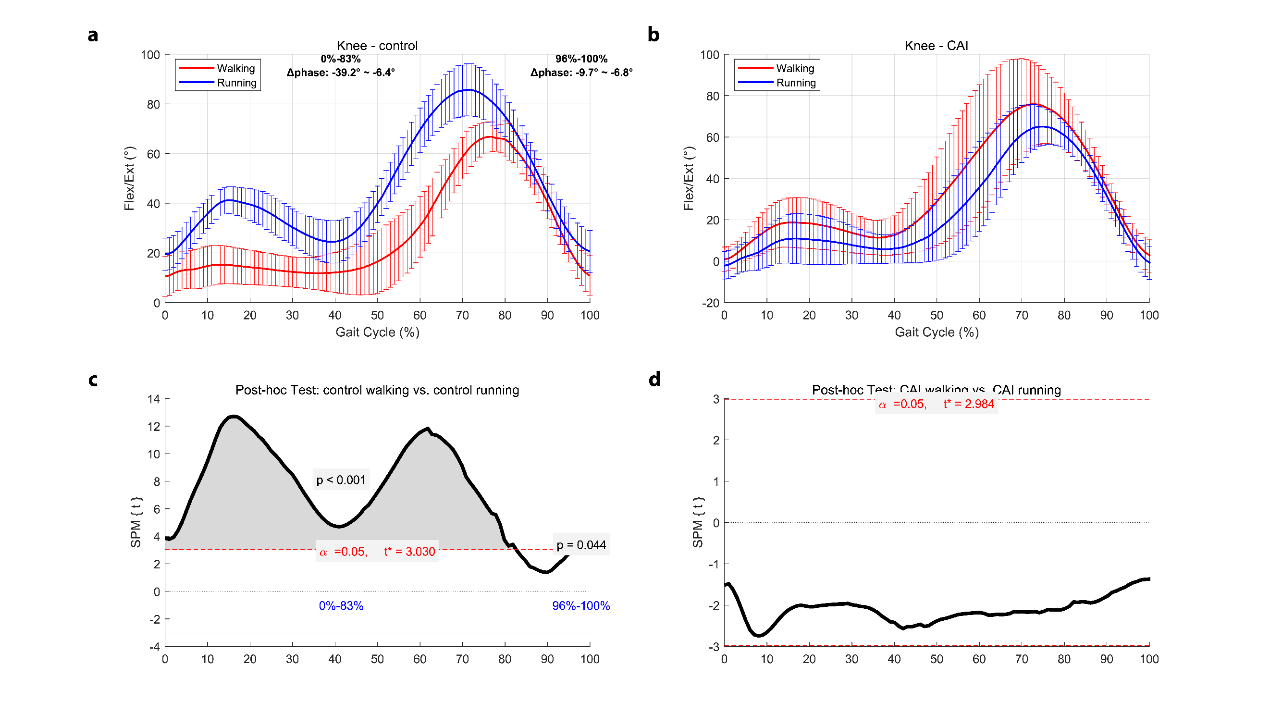


**Supplement_Figure4_Knee_GroupCompare.tif**

Comparison of knee flexion-extension between CAI and control groups across pooled gait conditions.

(a–b) Mean ± SD curves for CAI and control groups under combined walking and running conditions;

(c–d) SPM-t tests revealing significant intergroup differences.


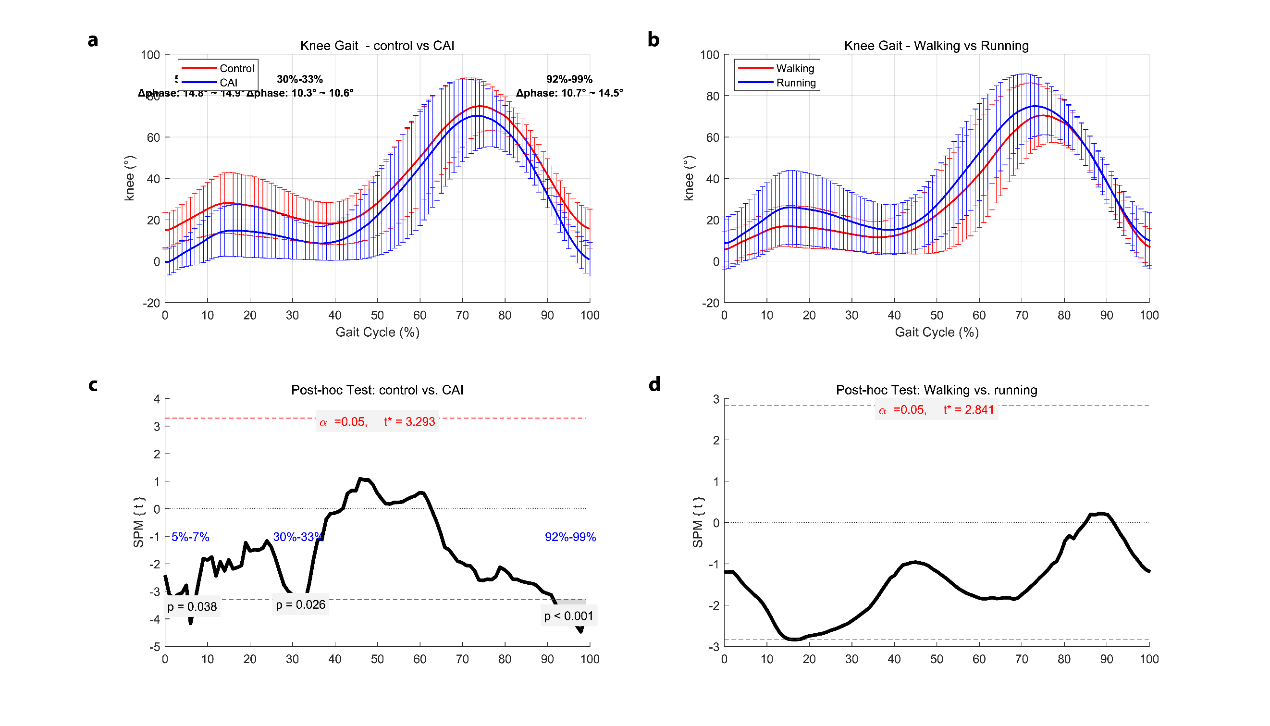


**Supplementary: Line 341**

**S1_Ankle_Frontal_Kinematics.tif**

Frontal plane ankle kinematics in CAI and control groups.

(a–c) Two-way SPM ANOVA results: (a) Group main effect (CAI vs. control), (b) Speed main effect (walking vs. running), and (c) Group × Speed interaction.

(d–e) Mean ± SD inversion/eversion angle curves for walking (d) and running (e) conditions in both groups.

(f–g) Post-hoc SPM t-tests for group differences under walking (f) and running (g) conditions.

This figure illustrates group differences in frontal plane ankle inversion/eversion angles between CAI and control groups. Two-way SPM ANOVA was used to evaluate main and interaction effects, and post-hoc comparisons were conducted for walking and running conditions.

**
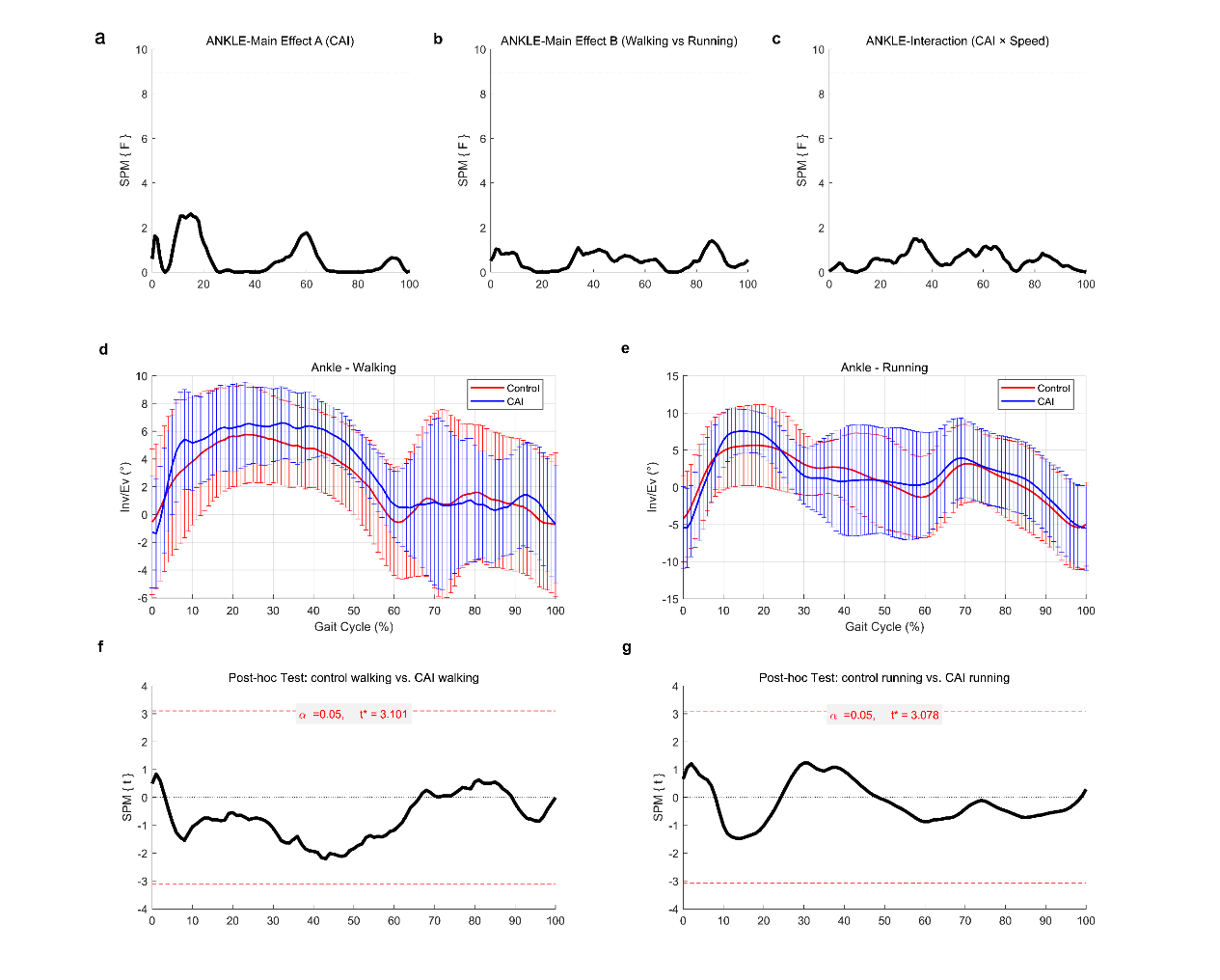
**

**S2_Ankle_Transverse_Kinematics.tif**

Transverse plane ankle kinematics in CAI and control groups.
(a–c) Two-way SPM ANOVA results: (a) Group effect, (b) Speed effect, (c) Interaction.
(d–e) Mean ± SD internal/external rotation angle curves during walking and running.
(f–g) Post-hoc SPM t-tests comparing CAI and control groups under each speed condition.

This figure presents transverse plane (internal/external rotation) ankle kinematics for both CAI and control groups, highlighting rotational stability differences under speed-dependent loading, based on SPM ANOVA and post-hoc results.


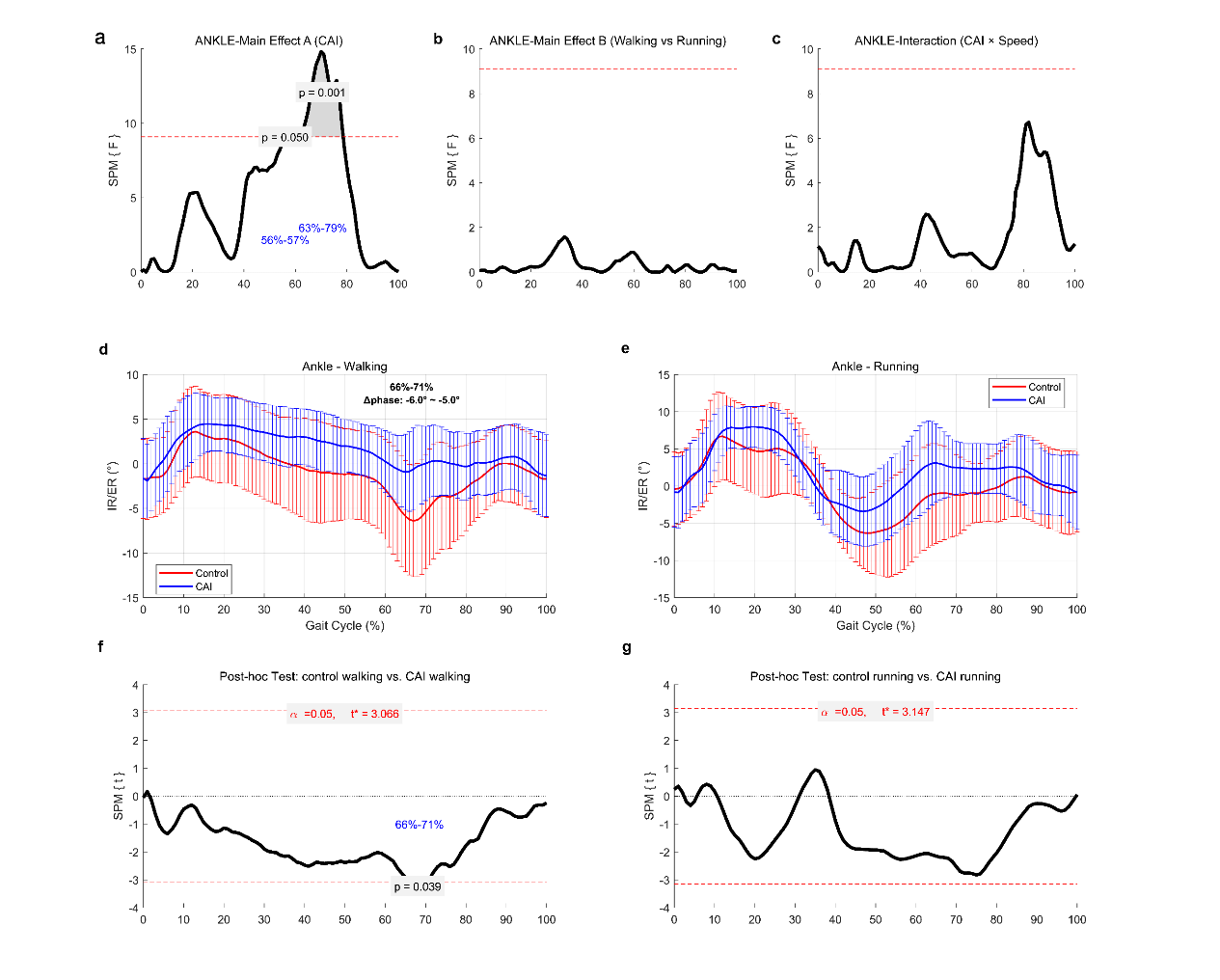


**S3_KS_AF_Vector_Coding.tif**

vector coding analysis of knee sagittal–ankle frontal (KS–AF) joint coordination.
(a–b) Mean joint angle trajectories for KS and AF.
(c–d) Vector coding angle curves and SPM-based comparisons between CAI and control groups.
Shaded areas indicate significant intervals with altered coordination patterns during walking and running.

This figure investigates inter-joint coordination between the sagittal knee and frontal ankle (KS–AF) planes using vector coding. The analysis highlights altered coordination patterns in CAI athletes, particularly during key gait phases.


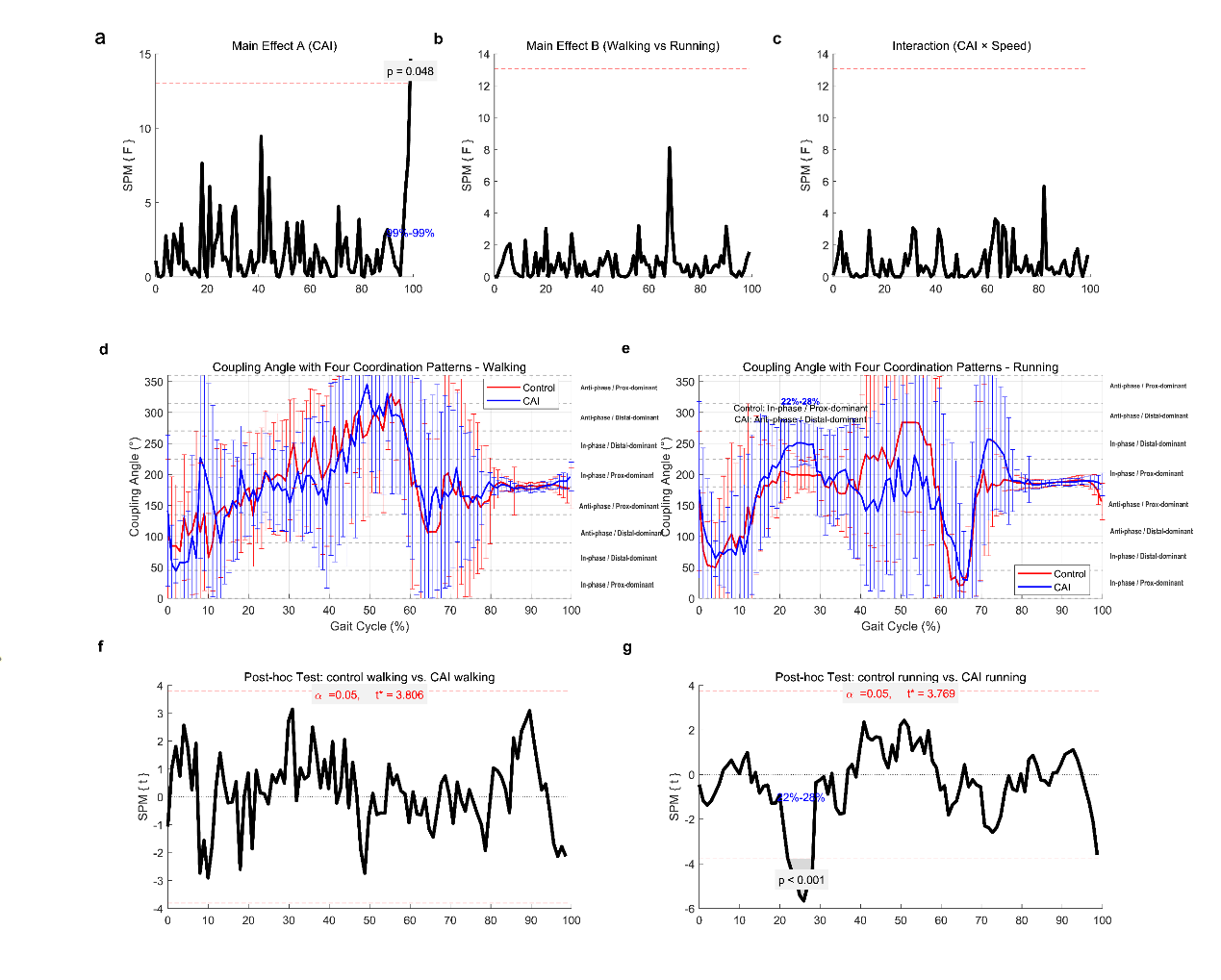


**S4_KS_AT_Vector_Coding.tif**

Vector coding analysis of knee sagittal–ankle transverse (KS–AT) joint coordination.
(a–b) Joint angle trajectories for sagittal knee and transverse ankle rotations.
(c–d) Vector coding curves and SPM comparisons showing group differences.
CAI group showed altered knee–ankle coupling during midstance and terminal stance phases.

This figure depicts coordination between sagittal knee and transverse ankle movements via vector coding analysis. The CAI group exhibited distinct coupling patterns, especially during midstance and late stance, suggesting compensatory adaptations.
